# Structural Diversity of Bacterial Communities Associated with Bloom-Forming Freshwater Cyanobacteria Differs According to the Cyanobacterial Genus

**DOI:** 10.1371/journal.pone.0140614

**Published:** 2015-11-18

**Authors:** Imen Louati, Noémie Pascault, Didier Debroas, Cécile Bernard, Jean-François Humbert, Julie Leloup

**Affiliations:** 1 *iEES-*PARIS, UMR 7618 (UPMC-CNRS-INRA-IRD-UPEC-Paris Diderot), UPMC, Paris, France; 2 Laboratoire Microorganismes: Génome et Environnement, UMR CNRS 6023, Clermont Université Blaise Pascal, Aubière, France; 3 MCAM MNHN, UMR CNRS 7245, Muséum National d’Histoire Naturelle, Sorbonne Universités, Paris, France; University of Freiburg, GERMANY

## Abstract

The factors and processes driving cyanobacterial blooms in eutrophic freshwater ecosystems have been extensively studied in the past decade. A growing number of these studies concern the direct or indirect interactions between cyanobacteria and heterotrophic bacteria. The presence of bacteria that are directly attached or immediately adjacent to cyanobacterial cells suggests that intense nutrient exchanges occur between these microorganisms. In order to determine if there is a specific association between cyanobacteria and bacteria, we compared the bacterial community composition during two cyanobacteria blooms of *Anabaena* (filamentous and N_2_-fixing) and *Microcystis* (colonial and non-N_2_ fixing) that occurred successively within the same lake. Using high-throughput sequencing, we revealed a clear distinction between associated and free-living communities and between cyanobacterial genera. The interactions between cyanobacteria and bacteria appeared to be based on dissolved organic matter degradation and on N recycling, both for N_2_-fixing and non N_2_-fixing cyanobacteria. Thus, the genus and potentially the species of cyanobacteria and its metabolic capacities appeared to select for the bacterial community in the phycosphere.

## Introduction

The global incidence and severity of cyanobacterial blooms have expanded during the past decades due to the eutrophication of many freshwater ecosystems and to climate warming [1]. Despite the increasing body of literature available on this phenomenon, it remains difficult to predict the onset, duration and intensity of blooms in a given ecosystem. This is in part due to a lack of knowledge concerning the processes leading to the dominance of cyanobacteria among the microbial primary producers and on the interactions between phototrophic and chemotrophic microorganisms [2]. Under certain environmental conditions cyanobacteria outcompete other phytoplanktonic microorganisms due to their high adaptive capacities for nutrient and light harvesting and their cellular organization in colonies or filaments [3]. In parallel to these traits the interactions between cyanobacteria and other bacteria have also been highlighted as a potential mechanism inferring a competitive advantage. The lower growth during the exponential phase of axenic *Microcystis aeruginasa* cultures as compared to non-axenic cultures [4] and the increase in nitrogen fixing (N_2_) capacities and growth in *Anabaena spp*. after addition of bacteria in cultures after addition of bacteria in cultures [5] suggests that these interactions might be important for cyanobacterial fitness.

As the presence of bacteria in cultures might have positive or negative effects on cyanobacterial growth (e.g. [6,7,8]) an in-depth description of the associated bacterial communities (i.e. phototrophic and chemotrophic bacteria other than the cyanobacteria) might permit a better understanding of the dynamics of cyanobacterial populations. Indeed, during cyanobacterial blooms bacterial cells can be directly attached to cyanobacterial cells (e.g. [9,10,5]) or can be associated in the surrounding area (e.g. [11]). In order to determine which bacterial species are found in the cyanobacterial phycosphere, i.e. the microhabitat where bacteria could be attracted by cyanobacteria (see [12]) the bacterial community (BC) has been examined during *Microcystis* blooms (e.g. [4,11,13,14]). These studies have suggested that the BC in the *Microcystis* phycosphere differed from free-living BCs in the same lake. Moreover, cyanobacterial species with different cellular organization (filament *versus* colony) could offer different microhabitats for bacteria [15] and the capacity of some cyanobacteria to fix N_2_ could also potentially influence the associated bacterial communities. For example, Hietanen et al [16] have shown that N_2_-fixing cyanobacteria supported heterotrophic growth particularly in nutrient deficient environments. Ploug et al [17,18] suggested (i) that the close association between cyanobacteria and heterotrophic organisms created a pH microenvironment that was advantageous for cyanobacterial iron uptake which in turn released fixed N compounds to the heterotrophic community, and (ii) that living on N_2_-fixing organisms could provide an advantage for non N_2_-fixing heterotrophic bacteria. From this work it appears that the metabolic capacities of cyanobacteria might influence the associated bacterial community. However, one of the main limitations of this work is the difficulty in separating the respective impact of the inherent bacterial diversity of the different lakes from that of the cyanobacterial bloom on the composition of the bacterial communities [19].

In order to assess the impact of the cyanobacteria bloom on one hand and the impact of the cyanobacterial genera on the other the BCs associated with two cyanobacterial blooms (*Anabaena* sp. and *Microcystis* sp.) that occurred successively within the same aquatic ecosystem were compared. This was also compared with the free-living BCs growing in the lake at the same time. A 454-pyrosequencing approach was performed on 16S rRNA amplicons using a combination of two different primer sets in order to minimize the proportions of cyanobacterial 16S rRNA sequences that overwhelmed the detection of other bacterial sequences as has been previously shown (e.g. [9,20]).

## Materials and Methods

### Study sites, sampling and fraction separation

The sampling site is a recreational lake located near the city of Champs-sur-Marne (Seine-et-Marne, Île-de-France, France, 48°51'47.0 N, 02°35'53.9 E). The lake has a surface area of 10.3 ha and an average depth of 2.70 m, and since 2005, has had several episodes of cyanobacterial blooms. This lake sampled is not a protected area and it's not located in a national park or connected with protection of wildlife. This studie did not involve endangered or protected species. All necessary permits were obtained from the Environmental and Biodiversity Policy services of the “Conseil Général” of Seine-Saint-Denis (Mrs Martine D'adda, Île-de-France, France,) to access the lake.

In 2012, two distinct cyanobacterial genera bloomed during summer (S1 Table). Water samples (>5 L) were collected during three consecutive days in July (bloom of *Anabaena*) and during three consecutive days in September (bloom of *Microcystis*). Each day, the water sample was filtered differentially to separate the free-living bacteria (ABF for *Anabaena* Bloom Free and MBF for *Microcystis* Bloom Free) and the cyanobacteria-associated bacteria (ABA for *Anabaena* Bloom Associated and MBA for *Microcystis* Bloom Associated) fractions.

For *Anabaena sp*., water samples were first filtered through a 20μm phytoplankton net in order to remove large detrital material and small eukaryotes that were very abundant in the samples. Then, 50 mL of filtrate containing a high density of *Anabaena* filaments were filtered through a 1.2 μm polycarbonate filter (Isopore Membrane Millipore) in order to concentrate ABA. The filtrate was then passed through a 0.2μm polycarbonate filter (Nuclepore Polycarbonate Whatman) in order to concentrate the ABF. For *Microcystis sp*., water samples were also filtered through a 20μm phytoplankton net, but due to the large size of the *Microcystis* colonies the concentrated material on the net was directly transferred to a cryotube for MBA. The remaining filtrate was then passed through a 0.2 μm polycarbonate filter (Nuclepore Polycarbonate Whatman) in order to concentrate the MBF. Samples were directly frozen in liquid nitrogen and stored at -80°C until DNA extraction.

### DNA extraction

DNA was extracted following the procedure of Massana et al [21] with some modifications. Briefly, each filter was flash-frozen in liquid nitrogen, and then transferred in Lysing Matrix E (MP Biomedicals, Illkirch, France) with 1.1 mL of lysis buffer (40mM EDTA, 50mM Tris-HCl, 0.75 M sucrose). Bead-beating was applied for 3x30 sec at a speed of 6.5 m s^-1^ (FastPrep®-24, MP Biomedicals, France). Then, Lysozyme (0.6 mg mL^-1^) was added to the filters and incubated at 37°C for 45 min with gentle stirring. Subsequently, sodium dodecyl sulfate (1% final concentration) and proteinase K (Thermo Scientific, France) were added, and incubated at 55°C for at least 90 min. Filter-debris were pelletized by centrifugation at 14 000 g for 5 min and supernatants were collected and purified twice by phenol-chloroform-isoamyl alcohol. After precipitation with sodium acetate (0.1 vol) and cold isopropanol (0.6 vol), the nucleic acids were washed with 70% ethanol, and then re-suspended in 100 μL milliQ water. The DNA was stored at -20°C until analysis.

### Pyrosequencing

The structure of BCs was assessed by pyrosequencing on gene coding for 16S rRNA. Two primer sets were used to amplify different variable regions on the 16S rRNA fragment. The first primer set, namely 563F [22] and 907rM [23], was used to amplify V4 regions of the major part of bacterial 16S rRNA fragments (including cyanobacteria). This primer set was named Eub-Pr1 for Eubacteria primer-set. The second primer set, namely 895F [24] and 1492R [25] was used to amplify the V6-V8 region across the bacteria excluding chloroplast and cyanobacterial sequences of the 16S rRNA fragment. This primer set was named NC-Pr2 for Non-Cyanobacteria primer set. *In silico* analyses against SILVA SSU Ref 108 NR database [26] confirmed that most of the cyanobacterial 16S rDNA sequences were excluded by NC-Pr2 and that some non-cyanobacterial sequences could be omitted (data not shown).

Ten base pair tags at 5’ position were added to primers to specifically identify each sample (given by Roche) together with adaptors: A Adaptor–TAGx –563F/895F and 907rM/1492R–B Adaptor. For each sample, three replicated PCR reactions (50 μl) were performed using the Phire Hot Start II DNA Polymerase (Fisher, France), with different optimized conditions. For the Eub-Pr1, the conditions are a first step of denaturation at 98°C for 3 min; 25 cycles of 94°C for 50 s, 52°C for 30 s and 72°C for 30 s; followed by 5 min at 72°C. For the primer set NC-Pr2, a touchdown round (without TAG) was preconized: 98°C for 3 min; 24 cycles of 98°C for 30 s, 65 to 55°C for 30 s with -0.4°C/cycle, 72°C for 60 s; followed by 12 cycles at 55°C and a final extension of 10 min at 72°C. A second round PCR was used to insert the TAG-primers within the sequences at an annealing temperature of 55°C.

The triplicate PCR products were pooled and purified using MinElute Gel Extraction Kit (Qiagen, Valencia, CA) following the manufacturer’s protocol and quantified using Qubit dsDNA HS Assay Kits (Invitrogen, France) following the manufacturer’s protocol. Pyrosequencing was carried out on 20 ng of amplicons per sample using a Roche 454 GS-FLX system (Titanium Chemistry) by GATC (Konstanz, Germany). A total of 24 PCR samples were pyrosequenced with 12 PCR samples per primer sets (6 for each bloom with 3 replicate per fraction). Only 22 samples were successfully sequenced on a half-plate run (1/4 run per primers set). Indeed, the same one replicate obtained for the Anabaena bloom failed in pyrosequencing making a total of 3 replicates for all samples except for ABA where two replicates were obtained for both primer sets.

### Clustering, alignment and phylogenetic of 16S rRNA gene fragments

A total of 180 876 reads (Eub-Pr1 primer set) and 276 806 reads (NC-Pr2 primer set) were obtained. The whole reads were analyzed with PANAM software: a tool for the Phylogenetic Analysis and Taxonomic Affiliation of SSU rRNA Amplicons (https://code.google.com/p/panam-phylogenetic-annotation/) [27]. All of these sequences were checked against the following quality criteria: (i) no Ns, (ii) quality score ≥ 23 according to the PANGEA process [28], (iii) a minimum sequence length of 200 bp and (iv) no sequencing error in the forward primer. The putative chimeras (UCHIME, [29]) and homopolymers [30] were detected. Secondly, the reads were de-multiplexed according to TAGx sequences in order to recover reads for each sample. Subsequently, reads were clustered in OTU (Operational Taxonomic Unit) with UCLUST [31] at a cut-off of 98% similarity. Then the OTUs were processed with USEARCH to obtain experimental reads sorted into different phyletic groups. Ad hoc profile alignments (*HMMER*, [32]) were processed prior building of maximum-likelihood phylogenetic trees (*FastTree*, [33]). Based on these trees an accurate taxonomy was assessed for each OTU. The reads assigned to cyanobacteria where removed from the data. Thresholds for abundant OTUs were defined with an abundance of reads > 1% within a sample [34].

In parallel, a BLAST analysis was performed on the representative sequences of abundant OTUs (> 1% in NC-Pr2 data) using the nucleotide collection nr/nt database. Sequences sharing at least a 98% sequence identity with the ones in GeneBank® were selected in order to determine their representativeness in this database and the ecosystems they were retrieved from.

### Statistical analyses

All the statistical analyses were performed using R software [35]. Normalization of sample size (number of reads per sample) was achieved by randomly resampling the same number of reads for each sample based on the smallest sample size.

Diversity indices were calculated on the normalized samples with “fossil” package [36] for Chao1 and ACE indexes and “vegan” package [37] for Shannon index. Differences in the diversity indices were determined by an Analysis of Variance (ANOVA). The average and standard deviations of these indices were then calculated for each bloom and fraction. The Venn diagram was constructed using the “VennDiagram” package [38] on three randomized and averaged selections at each time.

Differences in BCs composition between samples were statistically analyzed as following. A Correspondence analysis using the “ade4TkGUI” package [39] was performed to explore the relationship between bacterial OTUs obtained for each primer set used. Using the “vegan” package, a Mantel-test was performed to compare the Bray-Curtis-transformed OTUs distribution matrices obtained with Eub-Pr1 to that obtained with NC-Pr2.

Permutational Multivariate Analysis of Variance (PERMANOVA), carried out with the “vegan” package using Bray-Curtis distances matrices and 9999 permutations was used to analyze differences between BC at the OTUs level. Kruskal-Wallis tests were used with Bonferroni correction to compare the distribution of phylum/Class/Order between fraction and/or blooms.

### Nucleotide sequence accession numbers

The nucleotide reads determined in this study have been deposited to the SRA of EBI database with the accession number: PRJNA274325.

### Physical and chemical environmental variables

Water temperature and pH were determined with a multi-parameter probe (HI 98130, HANNA instruments). Chlorophyll-*a* (Chl*a*) content was determined by spectrophotometry. Total carbon and nitrogen contents were measured using a CHN Elemental Analyser (Elementar Vario EL III, Elementar Analysensysteme GmbH, Hanau, Germany). Phosphorous content was measured by the Olsen method [40]. Inorganic nitrogen compounds (N-NO_3_, N-NH_4_) were measured with a continuous-flow nitrogen analyser (SKALAR, San Plus System, Breda, the Netherlands).

## Results

### Eubacterial versus Non-cyanobacterial primers set

After cleaning, a total of 166 622 and 194 197 high quality reads were obtained for classical (Eub-Pr1) and Non-Cyanobacterial (NC-Pr2) primer sets respectively. As expected, the cyanobacterial reads dominated the Eub-Pr1 dataset with an average of 92% of the reads for the associated fraction and 32.7% for the free-living fraction (Table 1). The cyanobacterial reads from the Eub-Pr1 data were used to analyse the proportion of cyanobacteria for both bloom (S2 Table). The *Microcystis* reads and the *Anabaena* reads were the most abundant cyanobacteria for each bloom respectively (> 50% in the associated fractions). We also noted a less important presence of *Limnothrix* in the *Microcystis* bloom and *Uncultured Nostocales* in the *Anabaena* bloom. We also found some Uncultured cyanobacteria during both blooms

**Table 1:** Total cyanobacterial and non-cyanobacterial reads (mean ± standard deviation), observed bacterial richness and diversity indices obtained with both primer sets Eub-Pr1 and NC-Pr2. The average number of OTUs and diversity index were calculated after normalization to the smallest sample (n = 786 reads).

	Associated				Free-living			
	*Anabaena* bloom		*Microcystis* bloom		*Anabaena* bloom		*Microcystis* bloom	
	Eub-Pr1	NC-Pr2	Eub-Pr1	NC-Pr2	Eub-Pr1	NC-Pr2	Eub-Pr1	NC-Pr2
**Average no. reads**	12087 (±3227)	14386 (±3014)	16727 (±1932)	14073 (±4513)	17150 (±121)	18079 (±4600)	13606 (±2451)	22990 (±9537)
**Proportion of cyanobacteria reads (%)**	92.7	66.9	92.1	65.6	16.3	1.8	49.2	35.7
**Average no. reads without cyanobacteria**	883 (±135)	4757 (±1301)	1323 (±201)	4846 (±2006)	14357 (±3640)	17754 (±4688)	6917 (±3313)	14774 (±4110)
**No. OTUs**	94 (±20)	207 (±16)	59 (±8)	132 (±25)	85 (±9)	203 (±17)	117 (±5)	271 (±13)
**S**_**chao1**_ **estimate**	186.7 (±70.2)	456.3 (±116.6)	122.1 (±56.7)	293.7 (±57.5)	156.6 (±7.5)	455.7 (±33.4)	159.6 (±11.4)	705.5 (±32)
**S**_**ACE**_ **estimate**	190 (±75.1)	541.3 (±150.3)	125.2 (±45.8)	296.5 (±87.3)	144.7 (±11.6)	532 (±15.4)	163.4 (±14.3)	751.5 (±9.6)
**Shannon's diversity, *H***	3.1 (±0.8)	4.2 (±0.1)	2.4 (±0.2)	3.1 (±0.4)	2.9 (±0.6)	4 (±0.4)	4 (±0)	4.8 (±0.1)

In the NC-Pr2 dataset, cyanobacterial reads were less abundant with an average of 66.2% in the associated fraction, and 18.7% in the free-living one. Although NC-Pr2 was not fully selective against cyanobacterial 16S rDNA, this primer set decreased the proportion of cyanobacterial reads.

**Fig 1:**
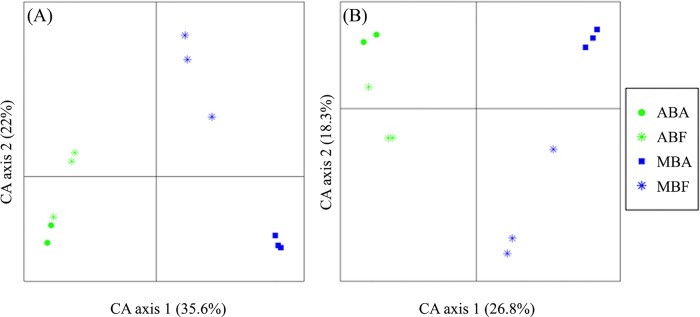
Correspondence analysis (CA) plot generated from the whole non cyanobacterial OTUs obtained with Eub-Pr1 (A) and NC-Pr2 (B) primer set, showing the distribution of the bacterial community after normalization to the smallest sample (n = 786 reads). (AB) for *Anabaena* bloom, (MB) for *Microcystis* bloom and (F) for free living fraction and (A) for the associated fraction.

After normalization (n = 786 due to the high proportion of cyanobacterial reads removed from Eub-Pr1 dataset) the same overall distribution was observed for both primer sets (Fig 1) when BCs were compared at the OTU level (Mantel-test; Pearson r = 0.97; *p*-value > 0.001). A clear discrimination of BC was observed between bloom-forming genus (*Anabaena versus Microcystis*) (first axis) and between associated and free-living states (second axis) (PERMANOVA; *p-*value = 0.01). Moreover, with both primers sets, BC distribution of *Anabaena* and *Microcystis* blooms were more distinct (PERMANOVA; *p-*value < 0.001) than free-living and associated bacterial communities during each bloom (PERMANOVA; *p-*value < 0.01) (Table 2).

**Table 2:** PERMANOVA comparing bacterial community composition obtained with Eub-Pr1 and NC-Pr2 primer set (n = 786). (Fraction) for the associated and free-living fractions and (Bloom) for cyanobacterial genera.

	*df*.		*F*		*R* ^*2*^		*Pr (>F)*	
	Eub-Pr1	NC-Pr2	Eub-Pr1	NC-Pr2	Eub-Pr1	NC-Pr2	Eub-Pr1	NC-Pr2
**Fraction**	1	1	5.712	8.497	0.205	0.228	**0.003**	**0.001**
**Bloom**	1	1	11.856	18.287	0.426	0.491	**< 0.001**	**< 0.001**
**Fraction × Bloom**	1	1	3.234	3.451	0.116	0.092	**0.024**	**0.03**

The estimated OTUs richness (Table 1) was higher in NC-Pr2 than Eub-Pr1 dataset (ANOVA; *p*-value < 0.001; Table 3). In the same way, the expected richness and diversity indices values (S.Chao and ACE values, Shannon index) were also significantly higher (ANOVA; *p*-value < 0.001; Table 3) with NC-Pr2 than with Eub-Pr1 dataset (Table 1).

**Table 3:** Comparison (ANOVA) of number of reads, number of OTUs and diversity indices after normalizing to the smallest sample (n = 786 reads). (Fraction) for associated and free-living fractions, (Bloom) for the cyanobacterial genus and (Primer) for the primer set used.

	OTU			Shannon's diversity, *H*			S_Chao1_			S_ACE_		
	*df*.	*F value*	*p-value*	*df*.	*F value*	*p-value*	*df*.	*F value*	*p-value*	*df*.	*F value*	*p-value*
**Primer**	1	319.526	**<0.001**	1	30.9386	**<0.001**	1	226.2705	**<0.001**	1	222.9428	**<0.001**
**Fraction**	1	64.305	**<0.001**	1	23.2948	**<0.001**	1	28.3885	**<0.001**	1	24.4063	**<0.001**
**Bloom**	1	0.315	0.583	1	1.0532	0.322	1	0.8114	0.382	1	0.0106	0.919
**Fraction × Primer**	1	13.451	**0.002**	1	0.1500	0.704	1	23.6131	**<0.001**	1	23.2685	**<0.001**
**Bloom × Primer**	1	0.025	0.874	1	1.0995	0.312	1	4.5612	**0.05**	1	0.3838	0.545
**Fraction × Bloom**	1	65.192	**<0.001**	1	29.0416	**<0.001**	1	29.6825	**<0.001**	1	28.5886	**<0.001**
**Fraction × Bloom × Primer**	1	8.265	**0.012**	1	0.0147	0.905	1	15.3226	**0.001**	1	13.8121	**0.002**

**Fig 2:**
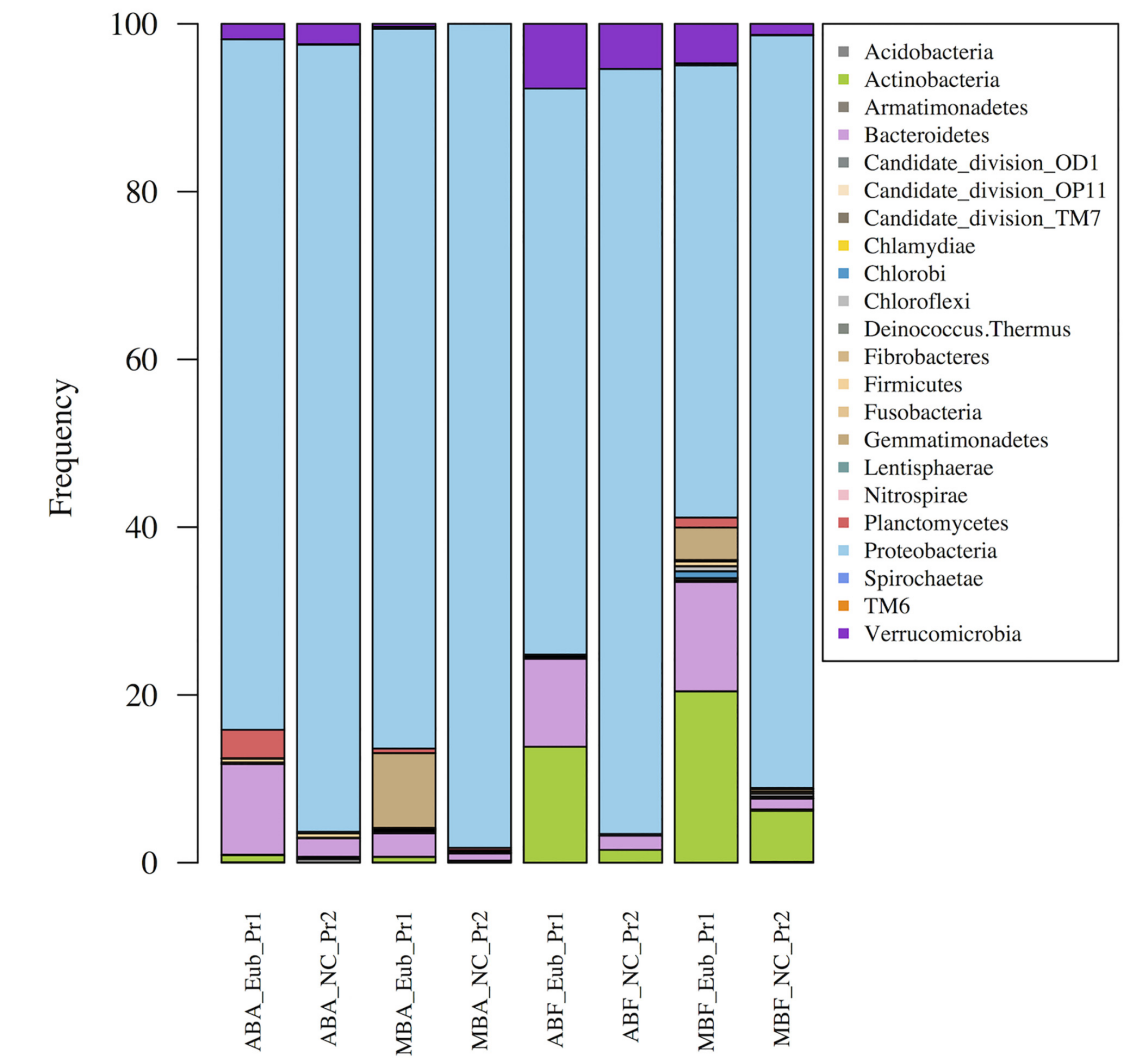
Relative abundance of the phyla obtained with Eub-Pr1 and NC-Pr2 primer set, expressed as the proportion of the average number of reads after normalization to the smallest sample (n = 786 reads). (AB) for *Anabaena* bloom, (MB) for *Microcystis* bloom, (F) for free living fraction and (A) for the associated fraction.

At the phylum level, all fractions were largely dominated by the Proteobacteria, followed by the Bacteroidetes (Fig 2), regardless of the primer set used. Similarly, the presence of Actinobacteria in the free-living fraction and their almost total absence in the associated fraction was observed for both primer sets. The only differences observed between the two primer sets were observed for rare phyla.

In conclusion, both primer sets provided similar results but knowing that the NC-Pr2 primer set (i) allowed a deeper BC description by decreasing the proportion of cyanobacterial 16S rRNA sequences and (ii) did not reduce the richness and diversity in the bacterial communities, the following in depth analysis was performed on data obtained with the NC-Pr2 primers.

### Comparison of bacterial communities of the *Anabaena* and *Microcystis* blooms

**Fig 3:**
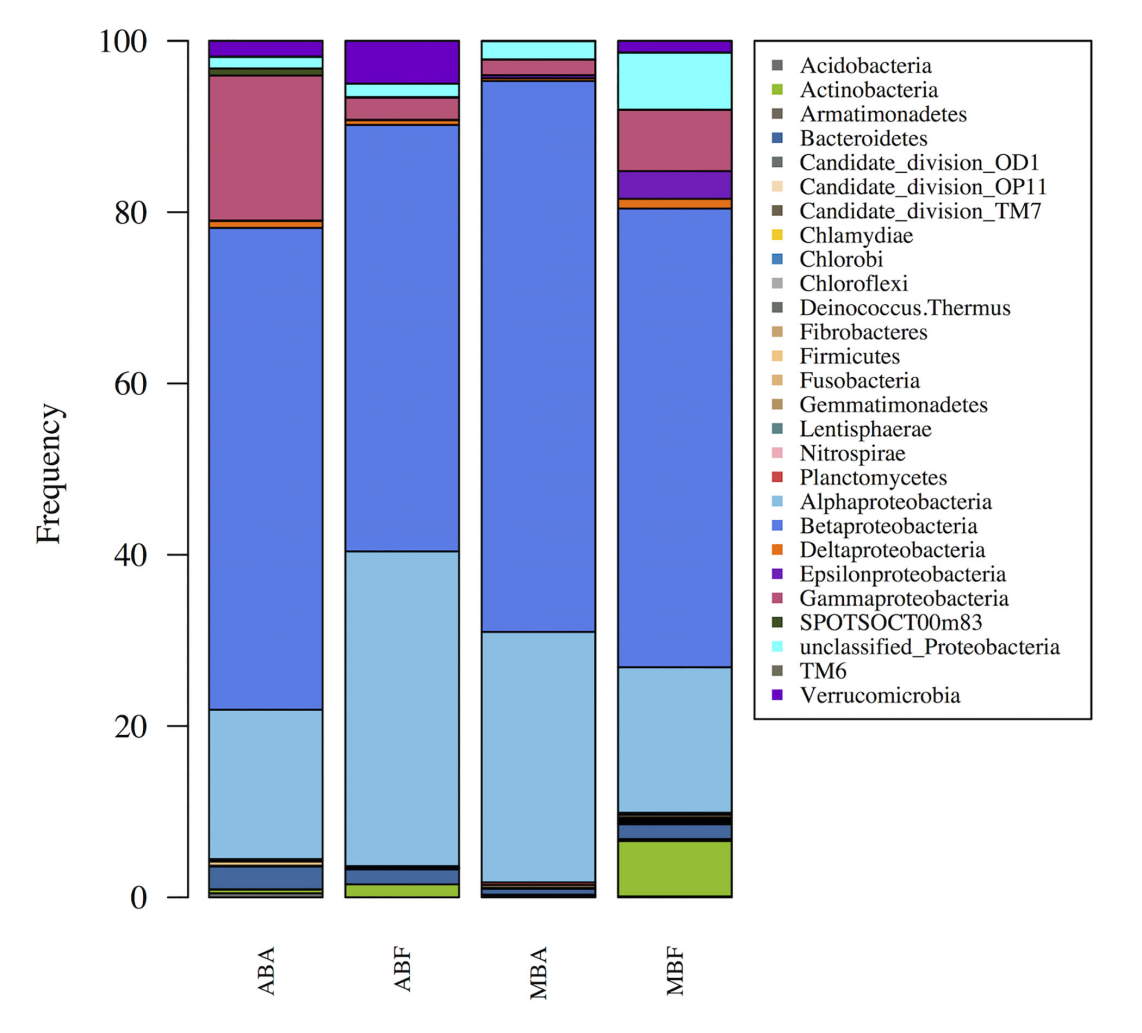
Relative abundance of the phyla and Proteobacteria classes, expressed as the proportion of the average number of reads from each sample after normalization to the smallest sample (n = 3402). (AB) for *Anabaena* bloom, (MB) for *Microcystis* bloom and (F) for free living fraction and (A) for the associated fraction.

After normalization all the bacterial communities at the phylum and class levels displayed a similar structure, characterized by the dominance of *Betaproteobacteria* and to a lesser extent, of *Alphaproteobacteria* and *Gammaproteobacteria* (Fig 3). Within the Proteobacteria phylum, *Betaproteobacteria* was always dominant regardless of the fraction and the bloom. On the other hand, the relative abundance of *Gammaproteobacteria* displayed significant variations within the *Anabaena* and *Microcystis* fractions (Kruskal-Wallis; *p-*value < 0.05). Finally, at the phylum level, the proportion of Actinobacteria was always higher in the free-living than in the associated fraction, and this was observed for both blooming cyanobacteria (Kruskal-Wallis; *p*-value = 0.01).

**Fig 4:**
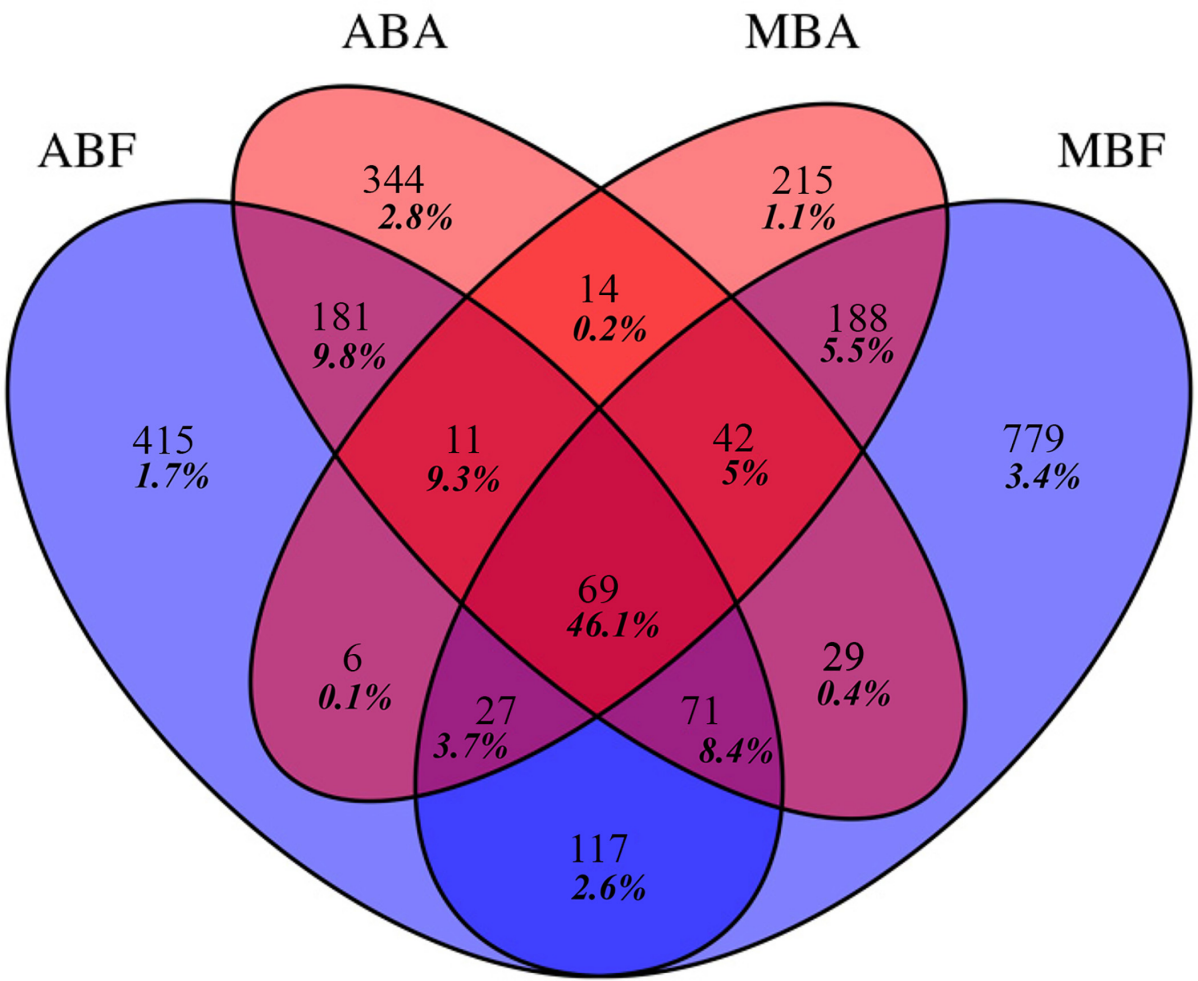
Distribution of the first 100 dominant bacteria OTUs at the order level, expressed as the proportion of the average number of reads obtained from each sample after normalization to the smallest sample. (n = 3402 reads). Circle size indicates the abundance relative to the whole sample for each cyanobacterial species: *Anabaena* (AB) and *Microcystis* (MB)) and fraction: free living (F) or associated (A) bacteria.

In contrast, at the OTU level (and also Family level, data not shown), the bacterial communities were clearly discriminated according to the cyanobacteria genus (*Anabaena* or *Microcystis*). As shown in Fig 4, the *Sphingomonadales* (Kruskal-Wallis; *p*-value < 0.01) and *Nitrosomonadales* (Kruskal-Wallis; *p*-value < 0.01) were notably abundant during the *Microcystis* bloom, while the *Xanthomonadales* (Kruskal-Wallis; *p*-value < 0.01) and *Chthoniobacterales* (Kruskal-Wallis; *p-*value < 0.05) were more abundant during the *Anabaena* bloom.

**Fig 5:**
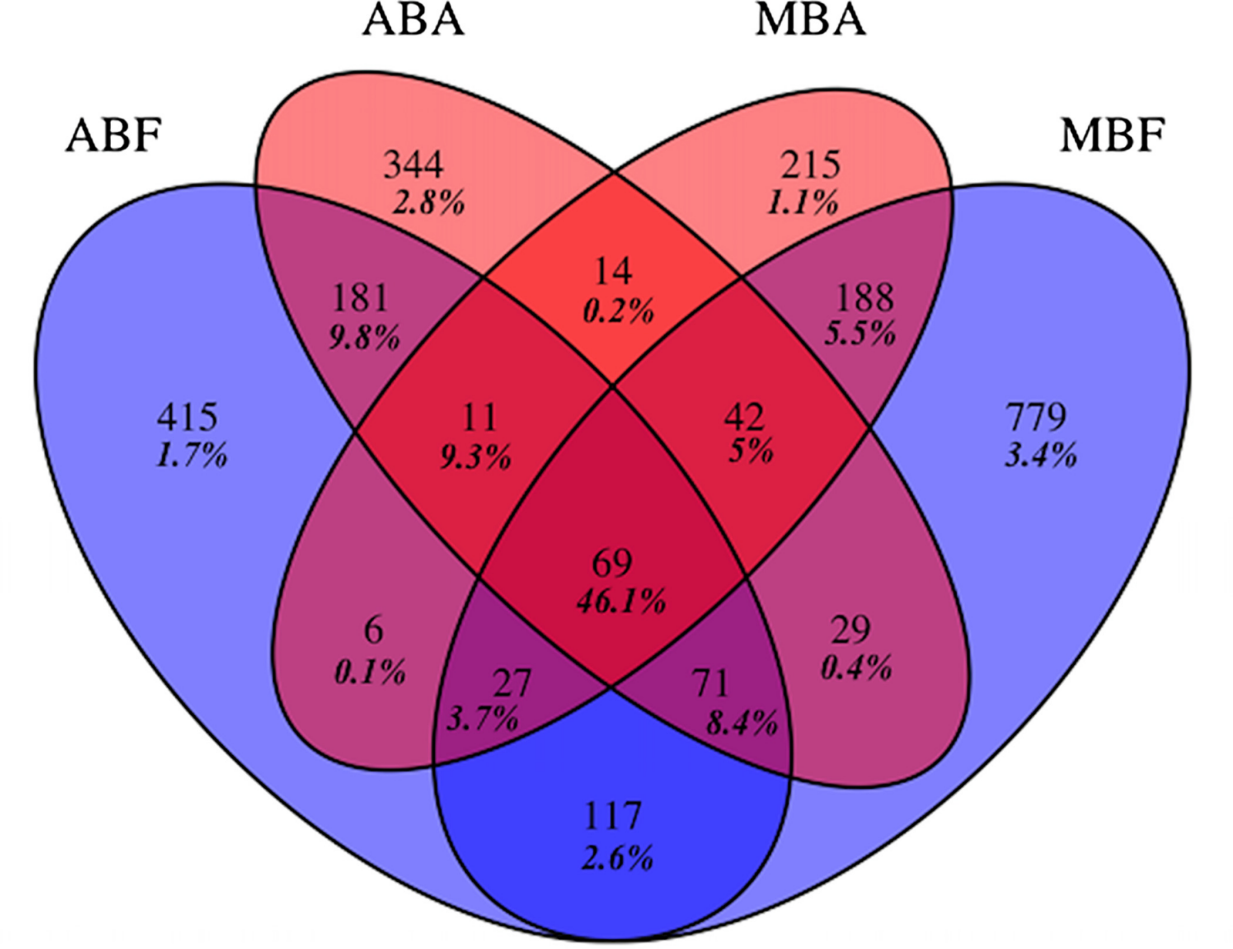
Venn diagram showing the unique and shared OTUs after normalization to the smallest sample (n = 3402 reads). (AB) for *Anabaena* bloom, (MB) for *Microcystis* bloom and (F) for free living fraction and (A) for the associated fraction. Relative proportions of those OTUs are shown in bold italic.

A low number of OTUs were shared by at least three fractions but these OTUs contained a very high number of reads (Fig 5). In contrast, a large majority of OTUs were found in only one fraction (ranged from 215 OTUs to 779 OTUs), however, they represented a very low number of reads (Fig 5). The number of OTUs shared by both free-living bacterial communities (117 OTUs) was more than five-fold higher than those shared by both associated bacterial communities (14 OTUs). This was also observed for the number of reads they contained (2.7 *versus* 0.2%).

The dominant OTUs (> 1%, 40 OTUs) accounted for 55.6% and 58.3% of the reads for associated and free-living fractions of *Anabaena* bloom respectively and for 73.1% and 40% of the reads for the associated and free-living fractions of *Microcystis* bloom respectively. The representative sequences of abundant OTUs were blasted to GeneBank® database (S3 Table) and around 10% of these OTUs displayed a sequence identity value < 98% with sequences from GeneBank®. Among the other OTUs displaying ≥ 98% sequence identity with sequences from GeneBank®, 37,5% of the OTUs sequences were found associated with cyanobacterial blooms and numerous sequences were retrieved in organic-matter-rich ecosystems worldwide (S3 Table).

## Discussion

### Primer set choice

In this study, a high-throughput sequencing approach was used to identify the bacterial taxonomic groups inhabiting the phycosphere of two cyanobacteria that bloomed successively in the same lake. In order to reduce the proportions of cyanobacterial reads that can represent more than 80% of the number of total reads (e.g. [9,11]) and to increase the proportion of bacterial reads, we tested a specific primer set (NC-Pr2). This set has been previously used to exclude chloroplast and cyanobacterial sequences [24]. Even if NC-Pr2 was only tested *in silico* (data not show) and was found to be exclusive to non-cyanobacteria species, it had, to the best of our knowledge, never been tested during a natural cyanobacterial bloom. Our findings have shown that the NC-Pr2 primer set offered the possibility of reducing the number of cyanobacterial sequences compared to that provided by a “classical” primer set (Eub-Pr1), despite the fact that they did not entirely exclude them.

The estimation of the composition of BCs depends on, for a substantial part, the choice of the sequencing strategy and of the targeted DNA region (e.g. [22]). But, when comparing data obtained by both primer sets, although they don’t target the same 16S rDNA regions, the same dominant phyla were retrieved and no major differences were found in the global structure of the communities and in the dominant OTUs. The NC-Pr2 primer set therefore can be used to compare BCs associated with bloom-forming cyanobacteria.

### Associated *versus* Free-living bacterial communities

We observed that the phyla diversity for the free living or associated BC could be positively or negatively affected, regardless of the cyanobacteria genus. The Proteobacteria dominated in both fractions, suggesting that this phylum is well adapted to occupy the phycosphere. Interestingly, Proteobacteria, and especially the *Betaproteobacteria*, were more abundant in the associated fractions. The *Betaproteobacteria* are known for their high efficiency in degrading dissolved organic matter (DOM) as shown by Niemi et al [41], and their abundance seemed to be positively correlated to low-molecular weight algally-derived substrates [42]. As the phycosphere is described as a hotspot for rapid cyanobacterial cellular turnover and organic matter fluxes (mainly fixed C and N photosynthetates and cellular C and N detritus) [2,43], this could explain their dominance in both fractions and their higher abundance in the associated fractions. Inversely, both associated fractions appeared to be depleted of Actinobacteria, a phylum that is generally found to be abundant in freshwater ecosystems [44,45,46]. However, the abundances of the Actinobacteria are also known to decrease in nutrient-enriched ecosystems [47] and to correlate inversely with cyanobacterial abundance, as shown by Ghai et al [48] and Parveen et al [49]. Thus, the Actinobacteria could be disadvantaged within the phycosphere due to the high bioavailability of DOM.

This suggested that within the phycosphere a specific selection of the bacterial consortium occurred *via* the release of cyanobacterial DOM. Thus, bacterial diversity appears to be driven by the metabolic capacity to degrade cyanobacterial exudates and detrital materials, regardless of the cyanobacterial genus. This assumption is also supported by the analysis of the distribution of the most abundant OTUs in GeneBank®, where they displayed high sequence similarities with various bacterial sequences generally retrieved in OM-rich ecosystems.

### *Anabaena* phycosphere *versus Microcystis* phycosphere

A clear distinction in overall BC structure between *Microcystis* and *Anabaena* blooms was observed, highlighting that each cyanobacterial genus promoted a specific BC within their phycosphere. This was observed for both the associated and free-living fractions status. Within the Proteobacteria, the proportions of *Gammaproteobacteria* were higher in the *Anabaena* phycosphere than in the *Microcystis* phycosphere, while the opposite was true for the *Alphaproteobacteria*. The latter were previously described as being abundant during bloom’s development (see for example [11,15,50]), however, the abundance varied according to the cyanobacterial growth status. The *Gammaproteobacteria* are usually found at very low abundances in freshwater lakes [51] and probably as a consequence of their copiotrophic metabolism, they are strongly enhanced in eutrophic conditions (e.g. [52]) and from beginning to decline of cyanobacterial bloom (e.g. [11]). It is known that quantity and quality of DOM produced during primary production depends on many factors including the species, physiological state and nutrient limitation [53]. Therefore it is probable that the DOM produced by cyanobacteria during the course of the bloom would vary in term of quality, quantity and, subsequently, bioavailability according to the species and the growth stage [54, 55]. Therefore, the different relative proportions of *Alpha-* and *Gammaproteobacteria* between *Anabaena* and *Microcystis* blooms could be due to different DOM qualities and quantities within the phycosphere of *Anabaena* and *Microcystis*, and as a consequence of the different sampling times (beginning of decline phase for *Anabaena* bloom and the exponential phase for *Microcystis* bloom, Table 4).

**Table 4:** Physicochemical measurements of lakewater for both cyanobacterial blooms. Chlorophyll-*α* content (Chl *α*), Nitrate (NO_3_), Ammonium (NH_4_), Dissolved Inorganic Nitrogen (DIN), Particular Organic Nitrogen (PON), Particular Carbon (PC), Dissolved Organic Carbon (DOC) and Particular Phosphorus (PP).

	Chl *α* (μg L^-1^)	NO_3_^-^ (mg L^-1^)	NH_4_^+^ (mg L^-1^)	DIN (mg L^-1^)	PON (mg L^-1^)	PC (mg L^-1^)	DOC (mg L^-1^)	PP (mg L^-1^)
*Anabaena* Bloom replicate 1	1689.2	0.13	0.17	0.3	7.6	44.4	10.2	0.07
*Anabaena* Bloom replicate 2	1502.6	0.28	0.29	0.57	16.2	55.2	16.1	0.11
*Anabaena* Bloom replicate 3	1344.9	0.1	0.29	0.39	17.6	77.9	18.5	0.09
*Microcystis* Bloom replicate 1	570.5	0.13	0.17	0.3	10.1	55.2	12.4	0.09
*Microcystis* Bloom replicate 2	368.2	0.11	0.3	0.41	8.2	41.0	11.5	0.04
*Microcystis* Bloom replicate 3	1591.4	0.09	0.18	0.27	na	na	13.9	na

Moreover, a high relative proportion of Verrucomicrobia occurred during the *Anabaena* bloom, supporting the hypothesis of a direct impact of the cyanobacteria on the bacterial communities. This phylum is known to be associated with high-nutrient environments or algal blooms [47,56]. Recently, Parveen et al [49] showed that cyanobacterial biomass could positively affect particle-associated Verrucomicrobial communities and that nitrate and inorganic nutrients could influence their dynamics [47,49].

In parallel, within the *Betaproteobacteria*, the proportion of *Nitrosomonadales* increased during the *Microcystis* bloom. Interestingly, this group encompasses bacteria involved in the nitrification (ammonia oxidation activity), one of the key steps for nitrogen recycling. The presence of this specific functional N-recycling group has already been highlighted in several studies, but only associated with N_2_-fixing cyanobacteria and during marine blooms and in the Baltic Sea for example, where no nitrification activity could be detected [57,58]. More recently, Ploug et al [18,59] have shown in the Baltic Sea that *Aphanizomenon sp*. and *Nodularia sp*. could release a high proportion of the photosynthates (35%) as NH_4_^+^. It is also known that during successive blooms of *Anabeana* and *Microcystis* [60,61], the fixed N pool could facilitate the following *Microcystis* bloom and thus enhance the functional groups involved in N-recycling.

Overall, we could hypothesize that cyanobacteria promote the diversity of their phycosphere BC based on their metabolic capacities for degrading their specific DOM. The phycosphere might also promote functional groups involved in N-recycling thereby supporting the mutualistic interactions between bacteria and N_2_-fixing and non N_2_-fixing cyanobacteria.

## Supporting Information

S1 Table Phytoplankton identification, counting and relative abundance species during the fist (*Anabaena* bloom) and second bloom (*Microcytis* bloom) (DOCX)

S2 Table Relative abundance of the cyanobacteria obtained with Eub-Pr1, expressed as the proportion (%) of the average number of reads from each sample after normalization to the smallest sample (n = 475). (AB) for *Anabaena* bloom, (MB) for *Microcystis* bloom and (F) for free living fraction and (A) for the associated fraction.
(DOCX)

S3 Table Blast analysis on the abundant OTUs per sample (>1%) after normalization to the smallest sample (n = 3402 reads). **(**F) for free living fraction and (A) the associated faction, (AB) for the *Anabaena* and (MB) for the *Microcystis* blooms. When the OTU sequence is shared at least 98% with more than 100 sequences in GeneBank®, the first 20 sequences were analysed.
(PDF)
