# Correction: Structural Diversity of Bacterial Communities Associated with Bloom-Forming Freshwater Cyanobacteria Differs According to the Cyanobacterial Genus

**DOI:** 10.1371/journal.pone.0146866

**Published:** 2016-01-07

**Authors:** Imen Louati, Noémie Pascault, Didier Debroas, Cécile Bernard, Jean-François Humbert, Julie Leloup

Fig 4, “Distribution of the first 100 dominant bacteria OTUs at the order level, expressed as the proportion of the average number of reads obtained from each sample after normalization to the smallest sample,” is a duplicate of Fig 5. Please view the correct Fig 4 here.

**Fig 4:**
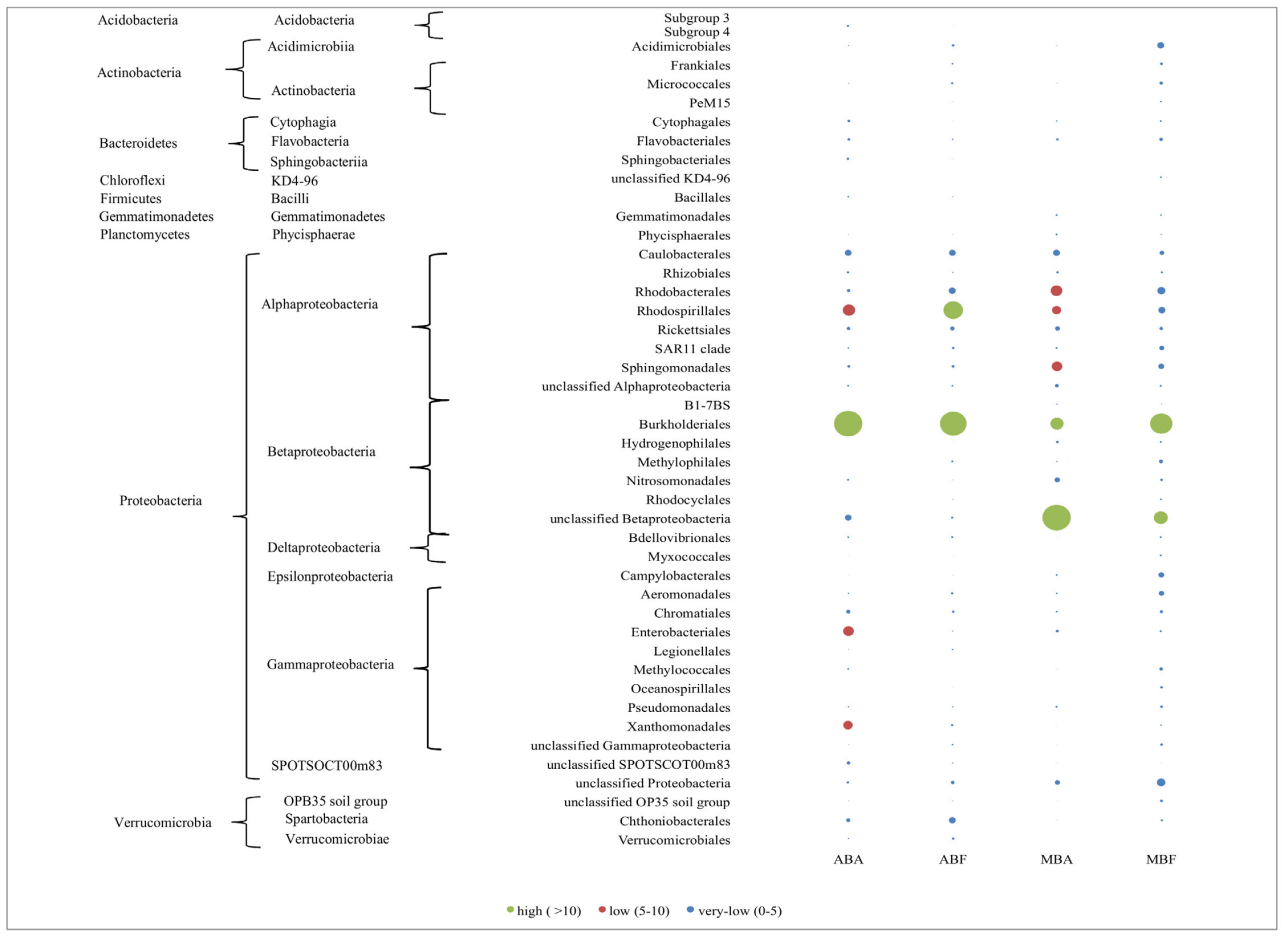
Distribution of the first 100 dominant bacteria OTUs at the order level, expressed as the proportion of the average number of reads obtained from each sample after normalization to the smallest sample. (n = 3402 reads). Circle size indicates the abundance relative to the whole sample for each cyanobacterial species: *Anabaena* (AB) and *Microcystis* (MB)) and fraction: free living (F) or associated (A) bacteria.
