# Supplementary material for: Structural Diversity of Bacterial Communities Associated with Bloom-Forming Freshwater Cyanobacteria Differs According to the Cyanobacterial Genus
Source: PLoS One. 2015 Nov 18;10(11):e0140614. doi: 10.1371/journal.pone.0140614 (PMC4651346; doi:10.1371/journal.pone.0140614)
Supplement: S1 Table — (DOCX) [file pone.0140614.s001.docx]

**S1 Table: Phytoplankton identification, counting and relative abundance species during the fist (*Anabaena* bloom) and second bloom (*Microcytis* bloom)**

| Sample volume | Order | Species | Number of Individuals | Relative abundance (%) |
| --- | --- | --- | --- | --- |
| First Bloom | | | | |
| 0.3L | Nostocales | Anabaena_bergii | 344 | 52.84 |
|  | Nostocales | Anabaena_sp | 4 | 0.61 |
|  | Nostocales | Anabaena_sp | 285 | 43.78 |
|  | Nostocales | Cuspidothrix_issatschenkoi | 1 | 0.15 |
|  | Chlorococcales | Kirchneriella_sp | 2 | 0.31 |
|  | Coscinodiscales | Melosira_granulata | 1 | 0.15 |
|  | Chlorococcales | Micractinium_pusillum | 6 | 0.92 |
|  | Naviculales | Nitzschia_sp | 2 | 0.31 |
|  | Oscillatoriales | Pseudanabaena_sp | 6 | 0.92 |
| Second Bloom | | | | |
| 0.5L | Chlamudomonadales | Chlamydomonas_sp | 1 | 0.03 |
|  | Desmidiales | Cosmarium sp | 1 | 0.03 |
|  | Chroococcales | Microcystis_aeruginosa | 2770 | 84.07 |
|  | Oscillatoriales | Pseudanabaena_mucicola | 522 | 15.84 |
|  | Chlorococcales | Scenedesmus_sp | 1 | 0.03 |
