# Supplementary material for: Structural Diversity of Bacterial Communities Associated with Bloom-Forming Freshwater Cyanobacteria Differs According to the Cyanobacterial Genus
Source: PLoS One. 2015 Nov 18;10(11):e0140614. doi: 10.1371/journal.pone.0140614 (PMC4651346; doi:10.1371/journal.pone.0140614)
Supplement: S2 Table — (AB) for Anabaena bloom, (MB) for Microcystis bloom and (F) for free living fraction and (A) for the associated fraction. (DOCX) [file pone.0140614.s002.docx]

**S2 Table: Relative abundance of the cyanobacteria obtained with Eub-Pr1, expressed as the proportion (%) of the average number of reads from each sample after normalization to the smallest sample (n = 475).** (AB) for *Anabaena* bloom, (MB) for *Microcystis* bloom and (F) for free living fraction and (A) for the associated fraction.

| Classification | | | ABA | ABF | MBA | MBF |
| --- | --- | --- | --- | --- | --- | --- |
| Subsection I (Chroococcales) | Family I | Microcystis | 0.11 | 0.35 | 91.86 | 64.63 |
|  |  | Synechococcus | 0 | 0 | 0 | 0.07 |
| Subsection II (Pleurocapsales) | FamilyII | Chroococcidiopsis | 0 | 0.35 | 0 | 0.07 |
| Subsection III (Oscillatoriales) | Family I | Limnothrix | 0 | 0 | 8.07 | 33.33 |
|  |  | Planktothrix | 0 | 0 | 0 | 0.07 |
|  |  | Unclassified | 0 | 0 | 0.07 | 0.07 |
| Subsection IV (Nostocales) | Family I | Anabaena | 57.79 | 22.67 | 0 | 0.07 |
|  |  | Aphanizomenon | 0 | 0.07 | 0 | 0.07 |
|  |  | Unclassified | 29.89 | 10.11 | 0 | 0 |
| unclassified Cyanobacteria | | | 12.21 | 66.46 | 0 | 1.62 |
