# Supplementary material for: Structural Diversity of Bacterial Communities Associated with Bloom-Forming Freshwater Cyanobacteria Differs According to the Cyanobacterial Genus
Source: PLoS One. 2015 Nov 18;10(11):e0140614. doi: 10.1371/journal.pone.0140614 (PMC4651346; doi:10.1371/journal.pone.0140614)
Supplement: S3 Table — (F) for free living fraction and (A) the associated faction, (AB) for the Anabaena and (MB) for the Microcystis blooms. When the OTU sequence is shared at least 98% with more than 100 sequences in GeneBank®, the first 20 sequences were analysed. (PDF) [file pone.0140614.s003.pdf]

S3 Table: **Blast analysis on the abundant OTUs per sample (>1%) after normalization to the smallest sample (n = 3402 reads). (**F) for free living fraction and (A) the attached faction, (AB) for the *Anabaena* and (MB) for the *Microcystis* blooms. When the OTU sequence is shared at least 98% with more than 100 sequences in GenBank™, the first 20 sequences were analyzed.

Taxonomy	% of reads ABA	% of reads ABF	% of reads MBA	% of reads MBF	# sequences ≥98% id	geographical origin	Originated ecosystem and function if it's identified	Associated or not to cyanobacterial bloom	Cyanobacteria associated	Accession number
**Proteobacteria; Gammaproteobacteria ; Enterobacteriales; Enterobacteriaceae**	1.05	0.09	0.01	0	> 100	China, India	Bacterial associated with fruit fly (2), rhizosphere (4), human feces (1), humain skin (+20),	not in the tested sequences	-	KM974659.1; AB920802.1; Q918061.1; JQ918057.1; KJ997976.1; KJ921705.1; KF516237.1; KJ803870.1; KJ718990.1; KJ718988.1; KJ718984.1; KF107414.1; KF107229.1; KF107143.1; KF107136.1; KF107135.1; KF107085.1; KF107080.1; KF107069.1
**Proteobacteria; Gammaproteobacteria ; Xanthomonadales; uncultured**	2.27	0.15	0.07	0	32	China, USA, Sweden	Lake Taihu (2), seawater (1), integrated lake epilimnion from pooled DNA from lake samples (3), Dongping lake (2), freshwater bacterioplankton during bloom (1), cyanobacterial bloom in freshwater pond (1), Tingxi freshwater reservoir (2), lake Gatun (2), Bantou freshwater reservoir (2), Lake Charles, Street and Industrial Canal (10)	yes	*Microcyctis aeruginosa*	JX406219.1; JN119212.1; HQ530659.1; FJ612377.1; AY509440.2; KC253327.1; JN869093.1; HQ661344.1; HQ661343.1; HQ530695.1; HQ530658.1; EU803605.1; EU803473.1; HQ661191.1; HQ661190.1; FJ354677.1; FJ353730.1; FJ349758.1; FJ349843.1; FJ350919.1
**Proteobacteria; Betaproteobacteria; Burkholderiales; Comamonadaceae; uncultured**	9.96	1.36	0.14	0.11	> 100	Germany, China, France, USA,	floodplain (1), Huangpu River (2), sludge in anoxic tank of hybrid reactor (1), activated sludge (6), municipal wastewater treatment plant (2), Biofilm (2), Wakulla Spring (1), root-associated bacteria in artificial wetlands (1), Hidou freshwater reservoir (1), freshwater calcareous mat (1), partial nitrifying-ANAMMOX municipal wastewater reactor (1), Anaerobic bioreduction of nitrate in hydroge-based membrane biofilm reactor (1)	not in the tested sequences	-	FM886840.1; JQ814744.1; JN391901.1; JN391885.1; F808871.1; HQ609628.1; EU283352.1; EF572731.1; CU466923.1; KJ013970.1; KF428047.1; KF836203.1; JX432005.1; JX431991.1; KC189638.1; JN204151.1; HQ661280.1; FJ484843.1; EF580972.1; HQ640616.1

**Proteobacteria; Betaproteobacteria; Burkholderiales; Comamonadaceae**	5.79	8.63	0.03	1.23	> 100	Netherlands, China, Japan, Germany, India, Singapore, France	Soil from Tet watersheds (2), lake Taihu (1), stream of Dianchi Lake (2), octylphenol polyethoxylate-degrading bacteria in river sediments (3), Dongping lake (2), river Leine sediment (1), iron-oxidation biofilm (2), freshwater biofilm (1), drinking water distribution system (1), low temperature hydrothermal oxides (1), early biofilms on different surfaces in urban freshwater (1), particle-associated betaproteobacetria in Lac du Bourget (1), Kelike Lake (1), low-grade copper bioleaching heap (1)	not in the tested sequences	-	JQ696279.1; JN869110.1; JF697516.1; AB374782.1; FJ612216.1; EU244077.1; AB252905.1; AB252904.1; KP071358.1; KF827196.1; JQ923990.1; JQ696326.1; JN860412.1; HQ860520.1; JF277873.1; HQ453932.1; AB374694.1; AB374686.1; HM129198.1; GU208313.1
**Proteobacteria; Betaproteobacteria**	0.06	0.82	34.21	6.40	5	China, USA	Lake Nanhu (1), lake Inbanuma (1), lake Mendota (1), 55-day-old anaerobic fermentation course of Microcystis blooms (1), enrichment sample(1)	yes	*Microcyctis*	JN941777.1; AB599922.1; FJ827947.1; GU559809.1; JQ958636.1
**Proteobacteria; Alphaproteobacteria; SAR11 clade**	0.01	0.03	0.10	1.36	> 100	USA, Chine, France, Germany, Japan, Canada	Mississippi River Basin (2), lake Niushan (1), Westensee lake water (1), deep mine drilling fluid (1), Lake Taihu (1), Miyu reservoir water (1), drinking water and granular activated carbon water (1), Green Lake (1), Lake Gatun (1), Delaware Bay (6), Chesapeake Bay (1), brackish samples from Weser estuary (1), Lake Kasumigaura (1), Pavin Lake (1), Coldspring Lake (1)	not in the tested sequences	-	KJ914640.1; JN941900.1; JX220031.1; JX448575.1; JN869020.1; GU305707.1; GQ453071.1; FJ916098.1; EU803454.1; EU801183.1; EU801142.1; EU800471.1; EU800443.1; EU800319.1; EU800141.1; EU800116.1; AY145598.1; AB154321.1; AY752103.1; KP687181.1
**Proteobacteria; Gammaproteobacteria ; Enterobacteriales; Enterobacteriaceae**	4.82	0.33	0.06	0.23	> 100	Portugal, China, India, Spain	Hospital effluents discharged in urban wastewater (1), sewage sludge domesticated by anaerobic-aerobic sequencing batch reactor (1), activated sludge in aerobic tank of urban sewage treatment plant (2), pathogens and normal flora of eel (6), digestive tract of freshwater fish (1), fermentation of idle batter (2), feces of *Euchoreutes naso* (1),human faeces (1), Jiulong River (1), water from Pichhola lake (1), gut microbiota from Scarab Larvae (2), pathogens from ornamental fish (1)	not in the tested sequences	-	LN624802.2; KM515974.1; KM391948.1; KM289145.1; KM289143.1; KM269016.1; KM269015.1; KM269014.1; KM269006.1; KM268972.1; KM268971.1; KM268970.1; KM222627.1; KJ830715.1; KJ830707.1; CP008841.1; KC812821.1; KJ660110.1; HG974539.1; KJ806341.1
**Proteobacteria; Betaproteobacteria; Burkholderiales; Comamonadaceae; Leptothrix**	3.92	0.15	0	0.02	> 100	USA, Denmark, China, Germany, Peru, New Zealand, Japan	Plant leaves in a freshwater (1), arsenite-oxidizing biofilm (1), active denitrifiers in activated sludge (3), Biofilm in a biofilter during phosphorus periodical bioaccumulation and harvesting processes (4), rhizosphere (1), embryonic stage of *Hydra vulgaris* (1), arctic stream sediment (1), Dongping lake sediment (3), periglacial soil (1), freshwater biofilm (1), activated sludge (2), Active denitrifiers in rice paddy soil (1)	not in the tested sequences	-	HM535419.1; AY168745.2; KF533783.1; JX431974.1; JN038694.1; HQ111167.1; FJ849351.1; GU208356.1; GU208297.1; GQ306142.1; FJ662745.1; KF533843.1; KF533789.1; JX431988.1; JX431971.1; JX431947.1; KC551610.1; KC551585.1; AB672130.1; HQ738453.1

**Proteobacteria; Betaproteobacteria; Burkholderiales; Comamonadaceae; Polaromonas**	0.13	0.73	2.42	2.21	> 100	China, Argentine, Antarctica, USA, Singapore, Spain, Indonesia	Lake Nanhu (1), Dongping lake (1), freshwater reservoir (1), lake Teganuma (1), Lundstrom lake (1), Lake Bosten (1) ,Biofilm-Electrode with Sulfur Autotrophic denitrification (1), *Paeonia ostii* rhizosphere soil (1), periphyton (1), activated sludge (2), ANAMMOX reactor (1), creosote polluted soil (1), sunflower rhizosphere (1), marine hot spring (1), industrial circulating cooling water system (1), activated sludge reactor (1), enrichment culture of grass carp intestine contents (1), primary drinking water supply (5), Yellowstone lake (1)	not in the tested sequences	-	JN941783.1; FJ612362.1; HQ008598.1; AB607278.1; FR691427.1; HQ436734.1; KP717532.1; KP663402.1; KM408583.1; KF712703.1; KF836351.1; JX257277.1; KC252871.1; AB516227.1; JX875876.1; JQ771995.1; JX047143.1; JQ323104.1; JN391672.1; JF830225.1
**Proteobacteria; Betaproteobacteria; Burkholderiales; Comamonadaceae**	2.57	4.94	0	0.01	> 100	China, Spain, United Kingdom, USA, Netherlands, Portugal, Iran	Shimen Realgar Mine (1), rhizoplane *Leontopodium alpinum* (1), floodplain soil (1), Zoige Alpine wetland (1), aerobic benzene-degrading consortium from benzene-contaminated groundwater from a sandstone aquifer (1), subsurface groundwater during polylactate stimulated chromate bioremediation (1), water treatment filters (1), uranium contaminated water and sediments (1), temperate grassland/deciduous forest (1), mine drainage water (2), sediment and groundwater with acetate amendment (6), subsurface aquifer sediment (1), water samples obtained from a micro saltwater lake (1), activated sludge from a membrane bioreactor (1)	not in the tested sequences	-	AB795550.1; AB681449.1; KC961265.1; JQ977158.1; JQ976511.1; JX222966.1; JX222618.1; HM151666.1; FJ801209.1; AM110076.2; DQ264511.1; EU130955.1; KF441648.1; KJ489982.1; KF975530.1; KF975527.1; JX225402.1; JX223352.1; JX222209.1; JX221781.1
**Proteobacteria; Alphaproteobacteria; Rhodospirillales; Acetobacteraceae; Rubritepida**	0	0.06	1.92	1.33	> 100	China, Panama, Greece, Germany,	55 and 60-day-old anaerobic fermentation course of *Microcystis* blooms (5), Dongping lake (1), lake Gatun (1), freshwater ponds (1), lake Nanhu (2),Dianchi lake (1), enrichment culture of grass carp intestine contents (1), Marathonas reservoir (2), freshwater bacterioplankton during cyanobacterial bloom (1), decaying chloramine residuals in a lab-scale system (4), lake Walchen (1)	yes	*Microcyctis, Aphanizomenon , Anabaena*	GU559835.1; FJ612228.1; EU803336.1; KC253364.1; JN941771.1; JN941770.1; HQ860513.1; JF830219.1; GU559849.1; GQ340312.1; AY509418.1; GQ340244.1; JQ629566.1; JQ629528.1; JQ629493.1; GU559800.1; GU559787.1; JF275008.1; JF830233.1; HQ532043.1
**Proteobacteria; Betaproteobacteria; Burkholderiales; Comamonadaceae**	0.92	1.79	0.02	0.10	> 100	Canada, Switzerland, Czech Republic, Spain, China, Sweeden, Finland, USA, Austria	Lake Ontario (1), epibionts of Daphnia sp. in lake Zurich (19), reservoir for drinking water supply (4), lake BassaOles (4), stream of Dianchi lake (5), Nam Co lake (3), Tanggulha lake (4) ,groundwater (1), lake Xinxinhai (1), groundwater from intermediate depth borehole (1), Chesapeake Bay (4), Lake Mondsee (1)	not in the tested sequences	-	KM031320.1; HF968602.1; HF968597.1; HF968596.1; HF968594.1; HF968591.1; HF968588.1; HF968586.1; HF968576.1; HF968572.1; HF968570.1; HF968569.1; HF968567.1; HF968565.1; HF968562.1; HF968559.1; HF968557.1; HF968552.1; HF968551.1; HF968550.1
**Verrucomicrobia; Spartobacteria; Chthoniobacterales; FukuN18 freshwater group**	0.89	2.43	0	0	15	USA, China, France, Japan	integrated lake epilimnion from pooled DNA from lake samples (6), lake Taihu (1), Pavin lake (2), lake Poyang (1), Bourget lake (2), Nam Co lake (1), Kelike Lake (1), methane oxidizing DHS reactor (2)	not in the tested sequences	-	HQ532160.1; HQ532044.1; HQ532023.1; HQ532021.1; HQ531561.1; HQ531550.1; AM690926.1; AY752089.1; AY752101.1; AF428656.1; JQ942016.1; HM129944.1; HM129219.1; AB504636.1; JQ942104.1

**Proteobacteria; Betaproteobacteria ;Burkholderiales; Alcaligenaceae; MWH-UniP1 aquatic group**	2.02	2.35	0.01	0	27	China, USA, Switzerland, Tanzania, Sweden, Panama, Greece, Germany, France	Sediments of lakes in Daqing (1), integrated lake epilimnion from pooled DNA from lake samples of Green Lake & Rock Lake (1), Bloom of *Planktothrix rubescens* in lake Zurich (1), Dongping lake (4), freshwater pond Dar es Salaam (2), lake Erken during cyanobacetria bloom (1),surface water of lake Niushan (1), carpet-like mucilaginous cyanobacterial blooms in a hypereutrophic lake (2), Shidou freshwater reservoir (2), Hubian freshwater reservoir (1), lake Gatun (2), Marathonas Reservoir (1), lake water (1), methanotrophic bacteria in Terrebonne Bay (1), source of drinking water (1), lake Poyang (1), *Microcystis* bloom in eutrophic bay (1), Changjiang river (1), composting of cow manure (1), drinking water treatment plant (1)	yes	*Planktothrix rubescens, Gloeotrichia echinulata, Microcystis*	KJ176840.1; HQ531231.1; FN668032.2; FJ612366.1; FJ612277.1; AJ565421.1; AY509453.2; JN941940.1; JF830210.1; HQ828025.1; HQ827932.1; HQ661266.1; HQ661227.1; EU803737.1; EU803579.1; EU037357.2; AJ565422.1; HQ661284.1; FJ612399.1; GQ340356.1
**Proteobacteria; Betaproteobacteria**	0.01	0.07	4.05	0.66	5	China, USA	Lake Nanhu (1), lake Inbanuma (1), lake Mendota (1), 55-day-old anaerobic fermentation course of *Microcystis* blooms (1), mucilaginous Cyanobacterial Aggregates (1)	yes	*Microcyctis*	JN941777.1; AB599922.1; FJ827947.1; GU559809.1; JQ958636.1
**Proteobacteria; Alphaproteobacteria; Caulobacterales; Caulobacteraceae; Phenylobacterium**	2.14	2.49	2.26	0.94	> 100	China, Greece, USA, Canada, Finland, Korea, Spain, New Zealand, Germany, Australia	Lake Kasumigaura (2), 60-day-old anaerobic fermentation course of *Microcystis* blooms (3), lake Kastoria (1), trichloroethylene-contaminated groundwater (1),anaerobic degradation of *Microcystis* blooms and surface sediment mixture (1), lake Xinxinhai (2), hypolimnion (hypoxic bottom) water from permafrost thaw pond (1), bacteria associated with Oxyria digyna (1), rhizosphere soil of eggplant (1), aquifier sediments (2), water of lotus flower pond (1), rhizosphere of maize and the surrounding carbonate-rich bulk soil (1), activated sludge (2), lake Grosse Fuchskuhle (1), bacteria in permafrost wetland (1), decaying chloramine residuals in a lab-scale system (1)	yes	*Microcyctis aeruginosa*	AB607297.1; GU559794.1; AF236003.1; AB607296.1; GU559797.1; GU559783.1; EF203203.1; AY706417.1; HQ904139.1; EU703406.1; JX221918.1; JN656835.1; EU703427.1; JX223018.1; HE814774.2; JF776893.1; FJ719086.1; FJ719043.1; KM035963.1; JN367066.1
**Proteobacteria; Alphaproteobacteria; Rhodospirillales**	0.80	1.72	0	0	4 (95%)	Germany	Oxic zone of 70-day-old flooded (unplanted) rice paddy soil microcosm (2), *Hydra vulgaris* (1), *Paulinella chromatophora* culture	not in the tested sequences	-	AM411930.1; AM411929.1; HQ111162.1; DQ789040.1
**Proteobacteria; Betaproteobacteria; Burkholderiales; Oxalobacteraceae; Massilia**	4.64	3.11	0	0.01	> 100	China, Italy, Germany, USA, Korea	Biofilm in freshwater (2), wastewater treatment plant (1), magnetite drainage sample (1), 1,2-dichloroethane contaminated aquifer (1), Xiao river (1), Arsenite oxidizing bacteria isolated from a goldmine (1), Chlorobenzene degrading bacteria isolated from a chlorobenzene-contaminated aquifer system (1), atrazine-mineralizing bacteria in wetland sediment (2), arsenic contaminated groundwater (2), petrochemcial wastewater treatment plant sludge (3), consortia of *Scenedemus obliquus* bacteria (2), health food drink (1), water of Giheung lake (1), solid-phase denitrification biofilm (1)	not in the tested sequences	-	KF827351.1; KF827067.1; JQ624270.1; HQ652596.1; FM204986.1; FJ230925.1; EU521706.1; AF235013.1; KP641171.1; LC015536.1; KP115663.1; KP115646.1; KF841311.1; KF841203.1; KJ782808.1; KJ782717.1; KJ782683.1; KJ632069.1; KJ660098.1; KF999729.1
**Proteobacteria; Alphaproteobacteria; Rhodospirillales**	6.80	19.32	0	0.02	3 (96%)	Germany, USA	Oxic zone of 70-day-old flooded (unplanted) rice paddy soil microcosm (2), *Paulinella chromatophora* culture (1)	not in the tested sequences	-	AM411930.1; AM411929.1; DQ789040.1

**Proteobacteria; Betaproteobacteria; Burkholderiales; Burkholderiaceae**	0.01	0.04	0.42	2.71	> 100	France, China, USA, Japan, Argentina	Rock pool filed with freshwater (1), freshwater ponds during cyanobacetrial bloom (1), surface water of lake Nanhu (2), industrial circulating cooling water system (1), Lake Taihu (2), enrichment culture of grass carp intestine contents (1), stream of Dianchi lake (1), Shidou freshwater reservoir (1), Yellowstone lake (2), surface of brackishwater, lake Hinuma (7), freshwater reservoir (1)	yes	not mentioned	NR_125545.1; KC253312.1; JN941782.1; JN941781.1; JQ323106.1; JN869087.1; JN869082.1; JF830207.1; JF697477.1; HQ661278.1; HM856538.1; HM856445.1; AB599874.1; AB599873.1; AB599872.1; AB599871.1; AB599870.1; AB599869.1; AB599868.1; HQ008538.1
**Proteobacteria; Betaproteobacteria; Burkholderiales; Burkholderiaceae; Burkholderia**	0	0.03	1.81	0.33	5 (95%)	China, USA	Lake Nanhu (1), lake Inbanuma (1), lake Mendota (1), 55-day-old anaerobic fermentation course of *Microcystis* blooms (1), mucilaginous Cyanobacterial Aggregates (1)	yes	*Microcytis*	JN941777.1; AB599922.1; FJ827947.1; GU559809.1; JQ958636.1
**Proteobacteria; Alphaproteobacteria; Rhodobacterales; Rhodobacteraceae; Rhodobacter**	0.01	0.03	1.33	0.51	1	China	Day 0 sample during anaerobic degradation of *Microcystis* blooms and surface sediment mixture	yes	*Microcytis*	HQ904129.1
**Proteobacteria; Betaproteobacteria; Burkholderiales; Comamonadaceae; uncultured**	6.52	0.53	0.02	0.04	> 100	Argentina, USA, Switzerland, China, Germany, France, Japan	Sphagnum moss from peat bog (1), arctic thermal spring (1), Chaerhan lake (1),sulfur containing freshwater source (2), activated sludge from membrane bioreactor (1), floodplain (1), Huangpu river (2), polycyclic aromatic hydrocarbons degrading bacterial community in retention systems which treat road runoffs (1), sludge in anoxic tank of hybrid reactor (2), activated sludge in a membrane bioreactor (2), sludge samples collected from biofilm or activated sludge (1), activated sludge (1), activated sludge of anaerobic/anoxic/aerobic process treating domestic wastewater (2), methane oxidizing DHS reactor (1), lake water isolated from algal microcosm (1)	not in the tested sequences	-	JF907240.1; JX257778.1; HM126838.1; AB476711.1; AB476707.1; EU283352.1; FM886840.1; KP280916.1; KP280898.1; AB980166.1; JQ814744.1; FQ658719.1; JN391901.1; JN391885.1; JF808871.1; HQ609628.1; HQ592570.1; AB543730.1; AB543729.1; AB504659.1
**Proteobacteria; Betaproteobacteria; Burkholderiales; Comamonadaceae; Variovorax**	0.42	1.01	0.03	2.08	> 100	China, Brazil, Thailand, Spain, Italy, USA, South Korea, Japan, Canada, Puerto Rico	Host of *Sogatella furcifera* (1), Endophytic bacteria in sugarcane wild germplasms (4), Endophytic bacteria and rhizosphere bacteria with cadmium-tolerance (1), Soil (1), biological soil crust of copper mine (2), citronella grass rhizosphere (1), acidophilic sulfate-reducing bacteria from Tinto River sediments (1),Variovorax bioemulsifiers producer (1), recirculating membrane reactor (1), contaminated soil (1), rhizosphere and bulk soil rich in potassium (1), poly(butylene sebacate)-degrading bacteria (1), bacteria growing at the expense of arbuscular mycorrhizal fungi (1), Bacterial Associations with weathered minerals (1), bacteria with carbonatogenic activity in consolidating treatment of deteriorated stone (1), parathion-degrading bacteria isolated from rice paddy soils (1)	not in the tested sequences	-	KP116945.1; KF557618.1; KJ200411.1; KJ185040.1; KJ185023.1; KJ184862.1; KF051781.1; JX575029.1; JQ770029.1; JQ769513.1; JN016600.1; JQ420060.1; JN627864.1; JN618391.1; JN561302.1; JF772527.1; AB627013.1; JF495126.1; FN668006.1; HQ445655.1

**Proteobacteria; Betaproteobacteria; Burkholderiales; Burkholderiaceae**	0	0.06	0.43	3.35	> 100	Austria, Switzerland, China, Japan, USA	Lake Mondsee (1), *Daphnia galeata* culture water (7), diseased leaf in lake Taihu (1), lake Taihu (1), lake Teganuma (1), lake Inbanuma (8), Dongjiang river (1), lake Kasumigaura (2), integrated lake epilimnion from pooled DNA from lake samples (1), lake Biwa (17)	not in the tested sequences	-	NR_125487.1; HF968620.1; HF968619.1; HF968617.1; HF968614.1; HF968612.1; HF968607.1; HF968606.1; JQ941855.1; JN868984.1; AB626845.1; AB626829.1; AB626824.1; AB626820.1; AB626819.1; AB626817.1; AB626816.1; AB626814.1; JF429291.1
**Proteobacteria; Betaproteobacteria; Burkholderiales; Comamonadaceae**	1.42	0.01	0	0	39	USA, Germany, China, Japan, New Zealand, Chile, Antarctica,	Arctic thermal spring (1), Chaerhan Lake (1), sulfur containing freshwater source (4), activated sludge (3), floodplain (1), Huangpu River (2), sludge in anoxic tank of hybrid reactor (2), activated sludge in a membrane bioreactor (2), sludge samples collected from biofilm or activated sludge (1), methane oxidizing DHS reactor (1), Lake Vida ice cover (2), batch anaerobic bioreduction of nitrate and sulfate in contaminated groundwater (1)	not in the tested sequences	-	JX257778.1; HM126838.1; AB476707.1; EU283352.1; FM886840.1; AB476711.1; KP280916.1; KP280898.1; JQ814744.1; JN391901.1; JN391885.1; JF808871.1; HQ609628.1; HQ592570.1; AB504659.1; EU104336.1; EU104142.1; EU104130.1; EF632937.1; EF572731.1
**Proteobacteria; Alphaproteobacteria; Rhodobacterales; Rhodobacteraceae**	0.42	2.63	0.01	0.14	> 100	Netherlands, USA, Sweden, China	Lake Taihu (3), water Column Lake Vinkeveense Plaasen (2), Kelike Lake (2), Wuliangsuhai Lake (3), Lake Zhaling (1), Chesapeake Bay (2), constructed wetland (1), Columbia River estuary (1), Delaware Bay (1), intestinal microflora in Chinese mitten crab (Eriocheir sinensis) (1), Lake Dongting (1), associated with cyanobacterial bloom (1), Dianchi Lake (1)	yes	*Aphanizomenon , Anabaena*	HQ904237.1; HQ904233.1; GU559760.1; JN869047.1; HQ904129.1; GU559790.1; AB515715.1; GU559808.1; FJ662739.1; EF095021.1; AF234745.1; HM128933.1; AY145582.1; DQ376570.1; KF427967.1; KC683251.1; KC683235.1; AB516210.1; HM773467.1; GU455339.1
**Proteobacteria; Alphaproteobacteria; Rhodobacterales; Rhodobacteraceae; Rhodobacter**	0.03	0.03	3.71	1.66	> 100	China, New Zealand, Germany,	Day 0, 55 and 60 sample during anaerobic degradation of Microcystis blooms and surface sediment mixture (4), 12, 55and 60-day-old anaerobic fermentation course of Microcystis blooms (3), Lake Taihu (1), polyhydroxyalkanoates accumulating organisms in activated sluge (2), freshwater biofilm (1), activated sludge from wastewater treatment plant (1), nitrifying-denitrifying activated sludge (1), Kelike Lake (1), brackish samples from Weser estuary (1), aerobic sequencing batch reactor (1), periphyton (2), biofilm-consortium (1), anaerobic fermentation reactor with waste activated sludge at thermophilic (1)	yes	*Microcyctis*	HQ904237.1; HQ904233.1; GU559760.1; JN869047.1; HQ904129.1; GU559790.1; AB515715.1; GU559808.1; FJ662739.1; EF095021.1; AF234745.1; HM128933.1; AY145582.1; DQ376570.1; KF427967.1; KC683251.1; KC683235.1; AB516210.1; HM773467.1; GU455339.1
**Proteobacteria; Betaproteobacteria; Burkholderiales; Comamonadaceae; Variovorax**	0.30	4.29	0.04	3.43	> 100	Canada, Switzerland, Spain, Netherlands, China, Czech Republic	Neuston biofilm in Lake Ontario and lake Ontario (2), epibionts of Daphnia in lake Zurich (3), Lake Filia (2), water columnof lake Vinkeveense Plaasen (2), Dianchi lake (4), bacetria in *Daphia magna* (1), lake Taihu (1), small deep pond (1), deep submontane lake in prealpine region (1), shallow fishpond (1), reservoir for drinking water supply (2)	not in the tested sequences	-	KP686749.1; KM105812.1; KM031328.1; KM031321.1; HF968603.1; HF968534.1; HF968511.1; HE857133.1; HE857081.1; HE655010.1; HE654968.1; HQ860537.1; JN392905.1; N868865.1; HE600689.1; HE600680.1; HE600678.1; HE600672.1; HE600668.1; HE600665.1

**Proteobacteria; Betaproteobacteria; Burkholderiales; Comamonadaceae; Polaromonas**	0.03	0.05	0.31	3.56	> 100	China, Czech republic, Japan, France, Austria	Streams of Dianchi lake (2), lake Taihu (1), reservoir for drinking water supply (1), lake Bosten (11), lake Ushikunuma (1), lake Inawashiro (1), particule associated *betaproteobacteria* in Bourget lac (1), Kelike lake (1), Chesapeake Bay (7), Delaware Bay (11), lake Inbanuma (1), Parker river (2), Ipswich river (1), lake Mondsee (3)	not in the tested sequences	-	KP687232.1; KP687208.1; KP687069.1; KP687065.1; KP686908.1; KP686891.1; KP686837.1; KP686827.1; KP686823.1; KP686822.1; KP686814.1; KP686804.1; KP686734.1; KP686636.1; KP686632.1; KP686589.1; HQ860581.1; JN868897.1; HE600685.1; JF429304.1
**Proteobacteria; Betaproteobacteria; Burkholderiales; Comamonadaceae; BAL58 marine group**	0	0.04	0.42	1.25	> 100	France, Switzerland, Canada, China, Japan, Spain, USA	Bourget lac (5), lake Zurich (83), lake Ontario (1), methane-oxidizing bacteria in a subtropical reservoir (1), lake Mizugaki (1), lake Llauset (1), lake Taihu (1), Dongjiang river (6), carpet-like mucilaginous cyanobacterial blooms in a hypereutrophic lake (2), integrated lake epilimnion from pooled DNA from lake samples (1), Yellowstone lake (4)	yes	*Microcyctis*	HQ453834.1; LN681434.1; KP687211.1; KP687122.1; KP686789.1; KP686775.1; LN827929.1; LN681518.1; LN681514.1; LN681510.1; LN681502.1; LN681494.1; LN681490.1; LN681482.1; LN681478.1; LN681474.1; LN681470.1; LN681466.1; LN681462.1; LN681450.1
**Proteobacteria; Alphaproteobacteria; Rhodospirillales; Acetobacteraceae; Roseomonas**	0	0	1.12	0.64	1	china	Kelike lake	not in the tested sequences		HM128975.1
**Proteobacteria; Alphaproteobacteria; Rhodospirillales; Acetobacteraceae; Roseomonas**	0.02	0.03	1.18	0.54	26	Australia, USA, Germany, China, Greece, Panama	Bacetria involved in decaying chloramine residuals in a lab-scale system (3), integrated lake epilimnion from pooled DNA from lake samples (1), lake Walchen (1), enrichment culture of grass carp intestine contents (2), 60 and 55-day-old anaerobic fermentation course of *Microcystis* blooms (3), Dongping lake (1), Marathonas reservoir (2), lake Gatun (1), freshwater of pond during cyanobacetrial bloom (1),lake Nanhu (2), Dianchi lake (1), freshwater bacterioplankton during bloom (1), Changjiang river (1)	yes	*Microcyctis, Aphanizomenon , Anabaena*	JQ629566.1; JQ629528.1; JQ629493.1; HQ532043.1; JF275008.1; JF830233.1; GU559835.1; FJ612228.1; GQ340094.1; EU803336.1; KC253364.1; JN941771.1; JN941770.1; HQ860513.1; JF830219.1; GU559849.1; GQ340312.1; AY509418.1; AF429252.1; GU559800.1
**Proteobacteria; Epsilonproteobacteria; Campylobacterales; Campylobacteraceae; Arcobacter**	0.01	0.03	0.03	1.47	> 100	China, Netherlands, India, Spain, Germany, china	sewage of Da Qing oil field (1), supernatant of activated treating municipal wastewater (9), wastewater treatment plat (1), Sewage (2), spinach-processing plant (11), lake Dianchi (1)	not in the tested sequences	-	DQ452478.1; LK393088.1; LK393086.1; LK393080.1; LK393078.1; LK393063.1; LK393047.1; LK393035.1; LK392994.1; LK392705.1; KC161908.1; HE565361.1; NR_117570.1; JQ845798.1; JQ845797.1; JQ845792.1; JQ845789.1; JQ845783.1; JQ845778.1; JQ845776.1

**Proteobacteria; unclassified Proteobacteria**	0.04	0.06	0.68	3.35	> 100	China, Switzerland, Spain, USA,	Lake Taihu (1), methylotrophic bacteria in Lake Zurich (10), Lake Llebreta (3), 'Eryuan' Hot Spring (1), Dongping lake (1), Lake Nera water column (1), Chesapeake Bay (4), Delaware Bay (6), Parker River (2), Biofilm (1), Wuliangsuhai Lake (1), Holocene marine sediment (1), reclaimed wastewater (1),	not in the tested sequences	-	JN869216.1; KP687204.1; KP686947.1; KP686884.1; KP686820.1; KP686715.1; LN794158.1; LN681526.1; LN681522.1; LN681527.1; LN681523.1; LN681532.1; LN681528.1; LN681524.1; LN681533.1; LN681529.1; KC886884.1; HE857485.1; HM184969.1; FJ612111.1
**Proteobacteria; Alphaproteobacteria; Rhodobacterales; Rhodobacteraceae; Rhodobacter**	0	0.01	1.81	0.75	20	China, Germany, United Kingdom, Canada, Spain, France	Day 0 and 55 sample during anaerobic degradation of *Microcystis* blooms and surface sediment mixture (4), 12, 55 and 60-day-old anaerobic fermentation course of *Microcystis* blooms (3), lake Taihu (1), Kelike lake (5), brackish samples from Weser estuary (1), wastewater treatment plant (1), lake Zhaling (1), lake Ontario (1), river sediment (1), lake Filia (1), mesophilic anaerobic digester which treats municipal wastewater sludge (1)	yes	not mentioned	HQ904129.1; HQ904237.1; HQ904233.1; GU559760.1; JN869047.1; GU559808.1; GU559790.1; HM128933.1; AY145582.1; HM129208.1; KC541086.1; FR774633.2; M128965.1; HM129032.1; EU703461.1; CU919934.1; KM031253.1; HE857093.1; KF963752.1; AB515715.1
**Proteobacteria; Betaproteobacteria; Burkholderiales; Comamonadaceae**	1.65	2.22	0.03	0	> 100	Hungary, USA, China, Italy, India, Japan, Singapore, Germany, United Kingdom, Iran	Biofilm developed in a hydrocarbon contaminated groundwater (1), loamy soil of Eucalyptus forest (1), freshwater (2), apple roots (1), drinking water distribution system (1), low temperature hydrothermal oxides (1), Dianchi lake (1), ferromanganese deposit (1), groundwater (1), early biofilms on different surfaces in urban freshwater (2), paddy soil from mining area (1), endosphere of hybrid poplar (1), drinking-water biofilm (1), polychlorinated biphenyl-polluted soil (1), oak root (1), mine drainage water (1), epiphytic bacteria in rivers impacted by mining (1), Lake Taihu (1)	not in the tested sequences	-	KP686576.1; LN613108.1; NR_074623.1; HQ118277.1; CP001013.1; X97070.1; L33974.1; KP686602.1; KJ014569.1; KF827196.1; KF799961.1; JX406232.1; JQ291763.1; JQ923990.1; JN860412.1; HQ860520.1; JN820188.1; AB602771.1; JF277873.1; FJ890906.1
**Proteobacteria; Betaproteobacteria**	0.01	0.28	8.93	2.65	4	China, USA	lake Nanhu (1), lake Inbanuma (1), lake Mendota (1), 55-day-old anaerobic fermentation course of *Microcystis* blooms (1)	yes	*Microcyctis*	JN941777.1; AB599922.1; FJ827947.1; GU559809.1
**Proteobacteria; Betaproteobacteria; Burkholderiales; Burkholderiaceae; Burkholderia**	0.02	0.03	2.12	0.48	4 (96%)	China, USA, Nigeria	lake Nanhu (1), lake Inbanuma (1), lake Mendota (1), hydrocarbon-contaminated soil (1)	not in the tested sequences	-	JN941777.1; AB599922.1; FJ827947.1; KF917023.1
**Proteobacteria; Alphaproteobacteria; Sphingomonadales; Erythrobacteraceae; Porphyrobacter**	0.04	0.35	6.22	1.65	> 100	Iran, China, Russia, United Kingdom, USA, Argentina, India, Arctic	Dialysis fluid (1), petrochemical wastewater treatment plant sludge (1), cyanobacterial mat (1), biofilm in a full scale experimental drinking water distribution system (1), active hydrothermal field (1), stromatolite from Socompa lake (1), constructed wetland (1), soil (1), endolith at an Arctic thermal spring (4), Wakulla Spring (1), tap water (6),biofilm of stone in lake Baikal (1)	yes	not mentioned	KP265965.1; KP265959.1; KP174470.1; KF964597.1; KM262803.1; KJ782740.1; KJ740149.1; KF611948.1; KJ549090.1; KJ187462.1; AB690710.1; HG008894.1; JX258056.1; JX257995.1; JX257980.1; JX257191.1; KC157047.1; KC189653.1; JQ906003.1; JQ906001.1
